# Consumer Attitudes Towards Health, Nutritional and Environmental Concerns of Beef Consumption by Confronting Different Information

**DOI:** 10.3390/foods15172984

**Published:** 2026-08-25

**Authors:** Sara Simunović, Igor Tomasevic, Blagoje Stojkovic, Mladen Rašeta, Aleksandar Bajcic, Stefan Simunovic, Ilija Djekic

**Affiliations:** 1 Institute of Meat Hygiene and Technology, Kacanskog 13, 11040 Belgrade, Serbia; mladen.raseta@inmes.rs (M.R.); aleksandar.bajcic@inmes.rs (A.B.); stefan.simunovic@inmes.rs (S.S.); 2 Department of Animal Source Food Technology, Faculty of Agriculture, University of Belgrade, Nemanjina 6, 11080 Belgrade, Serbia; tbigor@agrif.bg.ac.rs; 3 German Institute of Food Technologies (DIL), 49610 Quakenbruck, Germany; 4 Department of Animal Science, Faculty of Agriculture, University of Belgrade, 11000 Belgrade, Serbia; blagoje.stojkovic@agrif.bg.ac.rs; 5 Department of Food Safety and Quality Management, Faculty of Agriculture, University of Belgrade, Nemanjina 6, 11080 Belgrade, Serbia; idjekic@agrif.bg.ac.rs

**Keywords:** beef, health, nutrition, environment, consumers, information

## Abstract

The purpose of this study is to explore markets for fresh beef in Serbia by assessing consumers’ attitudes and awareness of three aspects, health, nutrition, and the environment, after being given information. Our working hypothesis was to explore the “halo” and “horn” effects of information about beef production and consumption on consumers. Surveys were carried out with a total sample of 956 beef consumers in order to analyze what they take into account in their buying decisions when they are directly informed. Consciously, we explored three different groups of consumers, consumers conscious about health, nutrition and the environment, which was confirmed after cluster analysis was done. The most numerous groups were customers conscious about health and nutrition, with 39.6% and 35.6% shares of the total number of consumers, respectively. Finally, our results show that consumers value health and environmental information the most, while they were not significantly affected by information about the nutritional benefits of beef.

## 1. Introduction

World meat production is predicted at 360 million tones (in carcass weight equivalent) in 2022, up by 1.2 percent in comparison to 2021 [1]. China is projected to account for most of the total increase in meat production, followed by Brazil and the United States. Such an increase in global meat production is led mainly by the growth in poultry production, while producers of other types of meat need to minimize the risk of greater declines in beef and pork production [1], especially when it is projected that global beef consumption will drop down by 5% by 2030 [2].

Meat and meat products contain important levels of protein, vitamins, minerals and micronutrients, which are essential for growth and development [3]. Furthermore, animal-sourced protein is part of a balanced diet, contributing valuable nutrients that are beneficial to health. Beef is associated with the definition of a “complete protein” because it contains all nine amino acids, in contrast to most plants [4]. Furthermore, when beef was compared to the other meat, species such as pork and poultry, volatile compounds were different [5]. Moreover, a lot of literature has been written to determine whether and how meat could affect human health [6]. Many authors who used health surveys and investigations to test the association between meat consumption and health outcomes revealed that excess intake of meat may cause cardiometabolic diseases and certain cancers [7,8,9].

However, although there are many studies analyzing different health, environmental, and nutritional impacts of food and meat consumption [6,8,9,10,11], there is a research gap when it comes to beef consumption. Furthermore, many studies focus on reducing meat consumption and supporting plant-based diets [12], but there is a lack of studies that explore how meat, especially beef, is essential nowadays in the human diet. Moreover, the influence of information on consumers’ concerns about health, the environment and nutrition was explored with limitations. There are a lot of studies emphasizing that providing information on the environmental impact and health consequences of meat consumption may reduce meat consumption and meat portion sizes [13], but there is a lack of research regarding both negative and positive health benefits, environmental and nutritional information and their “halo” and “horn” effects on beef consumers. This implies a need for studies like this and led us to our working hypothesis that was to explore the “halo” and “horn” effects of information about beef production and consumption on consumers.

Nowadays, it is well established that meat production presents a threat to the natural environment by contributing to climate change, resource depletion, and the extinction of species [14]. The food system as a whole accounts for an estimated 20–30% of global greenhouse gas emissions, while, within food consumption patterns, meat and dairy products are the main contributors to environmental impacts [15]. According to the previous literature, livestock (ruminant animals—cattle, goats, and sheep) produce methane through a process called ‘enteric fermentation’. The emissions of methane and nitrous oxide linked with livestock worldwide represented approximately 9% of total greenhouse gas (GHG) emissions in 2010 [16]. Furthermore, methane has a share of 16% of total global greenhouse gas emissions [17]. Additionally, goat production is associated with lower methane emissions compared to other domestic ruminants, which leads to the mitigation of climate change in red meat production [18]. In addition to livestock, other phases of meat production (slaughterhouses and meat processing plant) also have an impact on the environment [19]. This type of food consumption, especially meat consumption, is very popular nowadays, but the importance of the nutritional aspect also needs to be considered alongside other meat consumption aspects. Thus, many studies focus on dietary patterns and their adjustments for better human diet and lower environmental impact [11,20].

After the determination of these three aspects of meat production and consumption, there is one question left: “what do consumers think?”. The idea was to use surveys as a practical tool for gathering data from consumers with incorporated information about health, nutrition and the environment. The best solution for the information incorporation was the concept of a “preamble”. The function of preamble in our surveys is interpretive, in which the preamble is granted a guiding and informing role.

In contrast to the large number of studies analyzing consumers’ awareness about animal welfare or other sustainability attributes [10,21,22,23], only a few studies have segmented consumers based on their interests in the health, nutrition, and environmental aspects of meat production and consumption [24]. Many studies are concerned with only two aspects for further investigation, such as the influence of beef on environmental impact and nutritional quality of the diet, and environment and health benefits when meat was compared to meat alternatives [25,26]. However, there is a research gap in the literature on beef consumption and the “halo” and “horn” effects of information about beef consumption on consumers, including all three aspects: health, nutrition and the environment.

The main objective was to determine consumers’ profiles based on their attitudes associated with the perspectives, and to investigate whether direct information related to a specific perspective would affect consumers’ answers.

## 2. Materials and Methods

### 2.1. Questionnaire Design

The survey was made by using Google forms for making surveys (https://docs.google.com/forms/u/0/?pli=1, accessed on 18 August 2026). The questionnaire contained 15 questions, which were created based on the previous literature [24,27,28]. The questions were divided into four sections that included: (1) socio-demographic characteristics of the participants; (2) habits of beef consumption; (3) attitudes towards meat production and consumption their influence on health, nutrition and the environment; and (4) the importance of aspects. The same questionnaire was used in all four surveys differencing in the presence of a preamble (control survey does not contain preamble) and in the content of the preamble used. Preambles are given in Supplementary Material S1.

The preambles were created based on scientific, statistical and archival data from reliable literature. A total of three preambles were created, covering health issues, nutritional aspects, and environmental impacts associated with beef. The preamble about health concerns comprised results from the International Agency for Research on Cancer [29], and included some conclusions that certain consumptions of red meat may cause colorectal cancer. In parallel, information about antibiotic residues in meat that may lead to several adverse health effects, such as the development of multidrug-resistant microbial strains, allergic and anaphylactic reactions, and the disruption of normal intestinal flora, was provided. Furthermore, insights into diseases associated with meat, including both foodborne infections and intoxications, were presented at the end.

The preamble presenting health concerns was created to examine how consumers process health-related information. The information about meat is an extrinsic credence attribute, which depends on communication and trust in the information source. The variations in the quality evaluation of meat will be higher for taste, freshness and juiciness in comparison with credence quality dimensions such as healthiness and nutrition [30]. Nowadays, the concept of food quality has shifted from the safety, sensory and shelf-life aspects of food products to associations with nutrition, well-being and health [31].

When it comes to the preamble about the environmental impact of meat production and consumption, the hypothesis was that negative environmental information would determine consumers’ behavior and attitudes in a way that makes them more aware of this problem [32]. When the nexus among extrinsic attributes was made, it was found that consumers interested in origin of meat expressed high interest in health, price and environmental impact [24].

### 2.2. Experimental Design

An international web-based survey was conducted according to a similar study [24]. The questionnaire was validated by a small sample of nearly 12 researchers from the University of Belgrade to ensure the clarity, comprehensibility, and user-friendliness of the questionnaire [33]. The responses were about the information we were seeking, which was evidence supporting the validity of the questionnaire [34]. The surveys were carried out in the period from June 2022 to October 2022 throughout Serbia. In the same year, similar surveys were caried out among young Korean adults in their 20s [27]. Since dietary change can significantly contribute towards the achievement of the 2030 national and global sustainable development goal and meat consumption, our study, which provides data from 2022, is significant, as most previous studies have analyzed the consequences of dietary change prior to 2030 [11]. The participants represented potential Serbian beef consumers. One of the first questions served to distinguish and separate consumers who consume beef and ones who do not. the determination of a minimal number of respondents was made using sample size calculator (https://www.calculator.net/sample-size-calculator.html, accessed on 18 August 2026) [35,36]. According to this calculation, with a 95% confidence level, and a 5.57% margin of error, the proposed minimal sample size was 278. Thus, our planned sample size of 1000 respondents can be considered adequate [24].

### 2.3. Questionnaire Content

The first part of the survey collected information on consumer characteristics, including socio-demographic indicators and their habits in beef consumption. This information was important for further understanding the determinants of consumers’ attitudes and the importance of different aspects’ results. After eliminating all incomplete questionnaires, effective sample comprised 956 individuals (Table 1). A total number of incomplete surveys, for different reasons, was 10 unfilled by respondents. The excluded number of consumers was 34 as they responded that they “never” consume meat.

The second part of the questionnaire consisted of 20 statements covering the health, nutritional, and environmental dimensions associated with meat consumption, modified from the following publications [37,38,39,40]. For all statements, a 5-point Likert scale was used as follows: 1—totally disagree, 2—disagree, 3—neutral, 4—agree, and 5—totally agree.

Finally, in order to find out whether direct information influenced consumers, we analyzed consumers’ responses from the segment of the importance of aspects. Consumers were asked to assign a level of importance (1—“the most important” to 5—“the least important”) to each aspect.

### 2.4. Statistical Analyses

Exploratory factor analysis (EFA) was used to analyze all the statements. The EFA applied extracted seven factors. The correlation matrix was computed to determine the appropriateness of the factor analysis model. Bartlett’s test of sphericity was large and statistically significant (*p* < 0.05), suggesting that the data were suitable for factor analysis. The overall Kaiser–Meyer–Olkin measure was 0.81 and all individual KMO values that exceeded 0.6 were classified as ‘meritorious’; thus, we included these factors in the factor analysis [41]. Furthermore, Cronbach’s Alpha test was used to determine reliability (internal consistency) of each factor. All Cronbach’s Alpha values that exceeded 0.70 were accepted for further analysis [33].

Further, two-step cluster analysis was performed to reveal groupings within the dataset [42]. While usual classification uses predefined classes in which objects are assigned, clustering identifies similarities between objects first then makes groups according to characteristics in common [43]. For the purpose of the research, the two-step method determined three clusters as the optimal number of clusters according to characteristics in common. The clustering procedure was done with gender, age and education as categories, obtaining only attitudes extracted within factors from the EFA results. Consciously, we grouped these attitudes within three categories of consumers conscious about health, nutrition and the environment in order to explore if these will determine similar clusters. The main aim of cluster analysis was to confirm our working hypothesis regarding the influence of the three different preambles provided [20,44,45]. The differences between the segments were examined using the Kruskal–Wallis test. Kruskal–Wallis testing was employed to highlight significant differences between the clusters. Two Kruskal–Wallis tests with a 0.05 significant level were carried out to study the working hypothesis regarding the influence of the preambles provided.

To summarize whether there is a direct impact of information, and for which particular aspect, a chi-square test for goodness of fit was conducted (*p* < 0.05). This allows us to determine if differences in frequency exist across consumers’ responses. Thus, the null hypothesis, “the frequencies of the consumers’ answers about this aspect are not significantly different”, can be accepted or rejected. All statistical analyses were performed using the SPSS (SPSS 23.0, Chicago, IL, USA) package.

## 3. Results and Discussion

Factor analysis led to the results depicted in Table 2. These results show that the “risk of colorectal cancer”, “antibiotics’ residues”, and “pathogenic microorganisms” have the strongest influence on citizens’ health concern when consuming beef. The most positive evaluations result from “meat proteins’ positive effect” and a “great source of vitamins and minerals” within the group of nutritional variables. Finally, “greenhouse gas emissions” and “climate change” were revealed as the most significant aspects within the proposed environmental issues. According to the study of [33], the significance of heightened environmental attitude is more evident among consumers who have already reduced meat in their diet. Similarly, according to previous research, prior beliefs about the negative health and environmental impacts of meat consumption can moderate the intention to reduce meat intake [13,46,47,48]. These studies emphasized that environmental impact does not influence consumers to completely reduce meat consumption.

Three consumer clusters were divided. Cluster 1, ‘‘conscious about health” consumers (the largest cluster containing 39.6% of the sample), place a high emphasis on health care. This cluster is not equally distributed among demographic variables in the survey. Its members are predominantly women aged between 50 and 59 and with a PhD degree. Since they are better educated, and older, and more likely to be better informed about health issues, their concerns about health are higher than those in the other two clusters (Table 3). Furthermore, this cluster includes the highest percentage of the oldest eaters (60 and more). Similarly, in the study of [33], individuals were more receptive to information about the health and environmental impacts of meat consumption when it aligned with their preexisting beliefs. Furthermore, the study by [33] showed similar results for females, precisely among young adults who demonstrated a more positive impact on the intention to reduce meat consumption.

Cluster 2, ‘‘conscious about nutrition” consumers (35.6% of the sample), was a little weaker in its evaluations of health care when consuming beef. Opposite results were found in the study of [20], where both positive and negative nutritional impacts indicated that replacing beef with a combination of other foods without a significant effect on the nutrient profile of the diet is important and even a more feasible option of GHG emission mitigation. This cluster recognizes the demographic profile of the mostly male respondents within age group of 18–29 and consumers with a Bachelor’s degree. This cluster has the highest scores for “meat proteins’ positive effect” and “great source of vitamins and minerals” attitudes.

**Figure 1:**
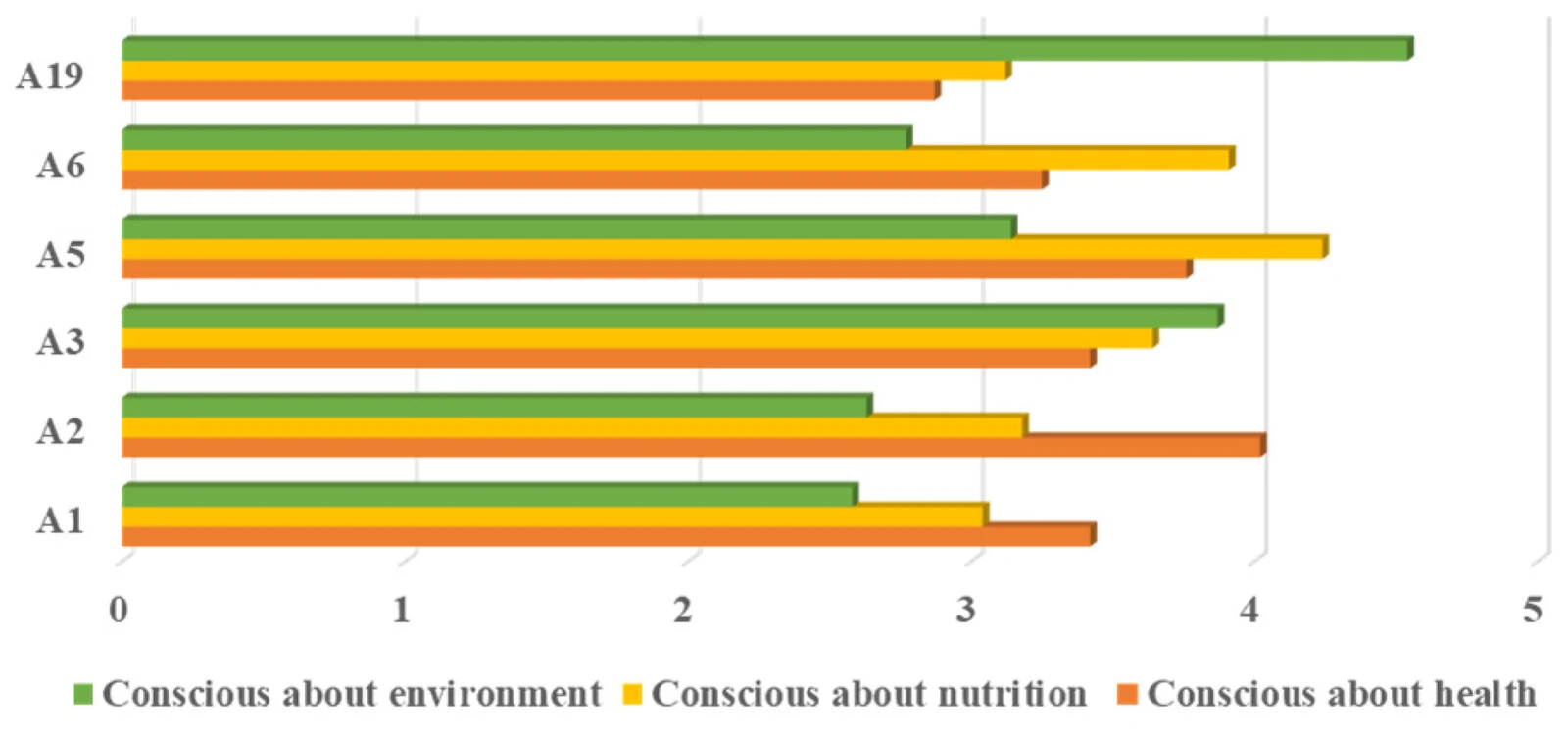
Presentation of attitudes through the scores obtained by clusters of consumers. Note: A numbers present following attitudes od consumers: A1: Moderately consumption of meat mitigates the risk of colorectal cancer; A2: It is important that meat does not contains antibiotics’ residues; A3: It is important that meat does not contains pathogenic microorganisms; A5: Meat proteins have great positive effect on human health; A6: Meat is a great source of vitamins and minerals; A19: Livestock is a great source of greenhouse gas emissions.

Cluster 3, “environmentally conscious” eaters (the smallest cluster containing 24.8% of the sample), puts most emphasis on the greenhouse gas emissions and climate change in livestock production. This is in accordance with study of [20], where the emissions of greenhouse gases (GHGs) and the carbon footprint were revealed as important in different real-life dietary patterns. The Green diet had almost the same carbon footprint as the Traditional and the Fast-food diets. This cluster has a high proportion of male respondents aged from 40 to 49, who finished career school. Cluster 3 also contains more men than the sample average, the second smallest percentage of highly educated people, and the lowest percentage of the oldest population. They hold a strong attitude towards reducing greenhouse gas emissions and the impact of livestock production on climate change (Figure 1).

### 3.1. The Relationship Between Consumers Attitudes and Clusters

According to the Kruskal–Wallis H test and chi-square values at the 0.05 significance level, differences between clusters were found (Table 3). The hypothesis that the distribution of consumers’ attitudes is the same across three clusters of consumers was rejected. The chi-square test indicated a significant (*p* = 0.05) association among the A1, A2, A5, A6, and A19 variables across all clusters, except A3 and A20 (Table 4). These results indicate that particular consumers’ attitudes significantly differ across the three clusters of consumers.

The first variable/consumer attitude that needs to be discussed is A1—“Moderate consumption of meat mitigates the risk of colorectal cancer”. This attitude significantly differs among clusters, where special attention should be paid to the mean value—the importance score of the attitude. Thus, the awareness of the mitigation the risk of colorectal cancer is the highest for cluster 1. The second strongly adopted attitude in the “conscious about health” cluster is A2, which refers to absence of antibiotic residues in meat. This strong adoption of this attitude can be related to well-known national monitoring of antibiotic residues in milk and milk products, meat and meat products, egg, feces and urine that is necessary to safeguard the health of the consumers, as well as minimize environmental contamination [49].

When it comes to Serbia, it is well known that beef is an essential part of the human diet. Since the mid-19th century, beef has been mostly consumed through soup, in the form of boiled beef. Many Serbian dishes and meat products include beef in their compositions [50]. On the other hand, dietary patterns in Denmark showed that there is evidence of reduced meat consumption [20]. Nowadays, the promotion of the nutritional benefits of reformulated meat products and meat analogues is in expansion [51].

Nutrition knowledge usually refers to the knowledge of concepts and processes related to nutrition and health benefits [52]. Thus, nutritional claims such as the “increased calcium” claim, “containing omega-3” claim, and “reduced salt” claim increased consumers’ willingness to pay for meat products [53]. In our study, cluster 2 is clearly divided, where consumers that are conscious about nutrition recognized that “meat proteins have a great positive effect on human health” and that “meat is a great source of vitamins and minerals”. According to other studies, nutritional knowledge can be improved among meat consumers by using the most prevalent technique: general nutrition education [54].

The environmental perspective of food systems is different in comparison with other production systems, due to the agricultural phase [55]. In food sector, there are differences between plant-based and animal-based food companies in health, nutrition, and environmental impacts globally [42,51]. Furthermore, in the study of [20], the environmental impact of different types of beef and the route from slaughterhouse to plate that affect the GHG emissions were emphasized, in accordance with the dietary demands of Danish consumers considering different existing dietary patterns.

In line with our results, European consumers’ awareness of the environmental consequences of intensive livestock production has been progressively growing [56], especially when it comes to greenhouse gas emissions, where the calculation of methane emissions noted a growing trend of 75% from 1994 to 2018 [57]. In the study of [58], segmentation of consumers was also done, where one group of consumers was more concerned about the environment and believed in the “consequential effect” of their choices.

### 3.2. Impact of Information

**Figure 2:**
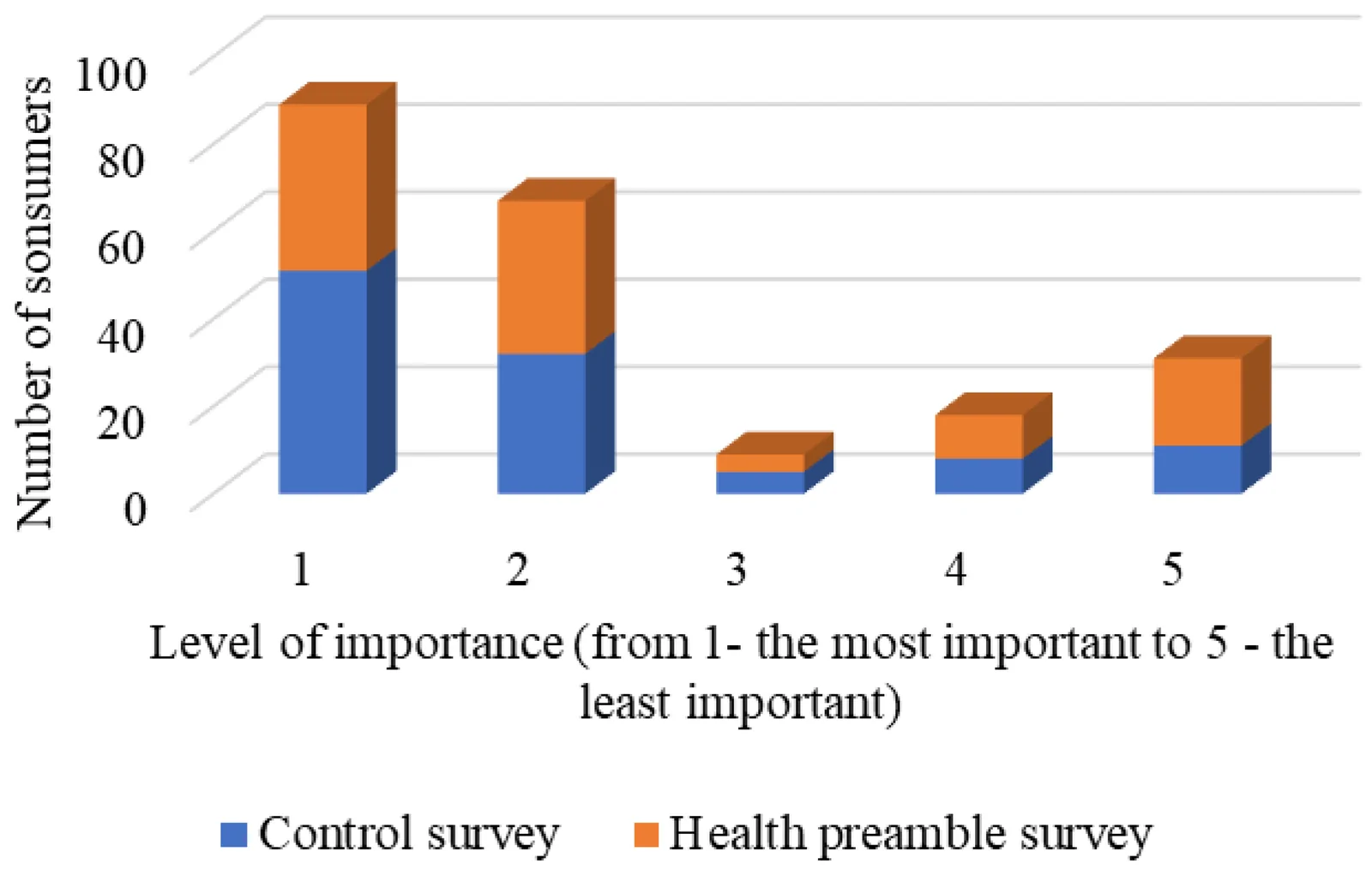
Consumers’ answers regarding the level of importance when comparing control and health surveys.

**Figure 3:**
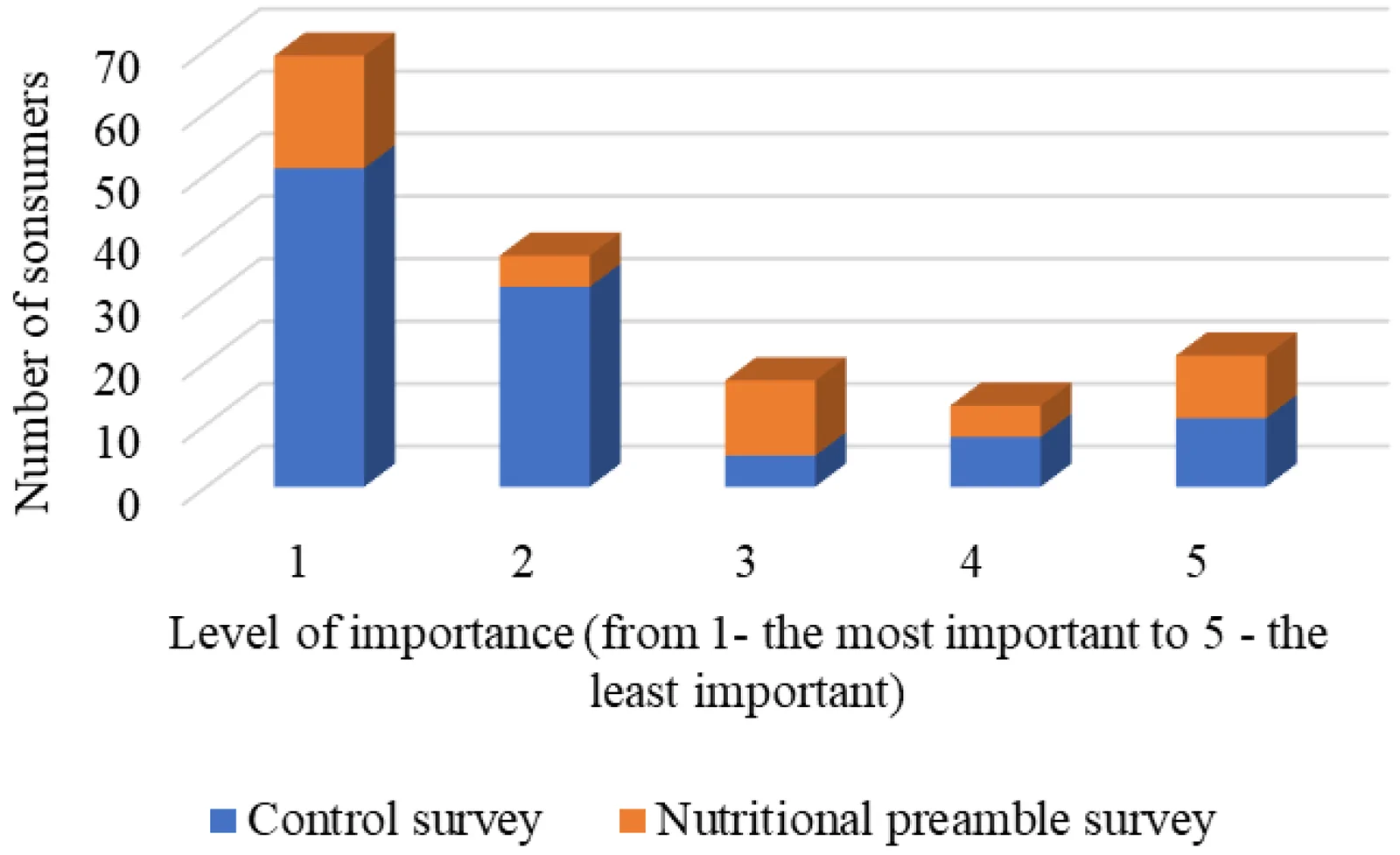
Consumers’ answers regarding the level of importance when comparing control and nutrition surveys.

**Figure 4:**
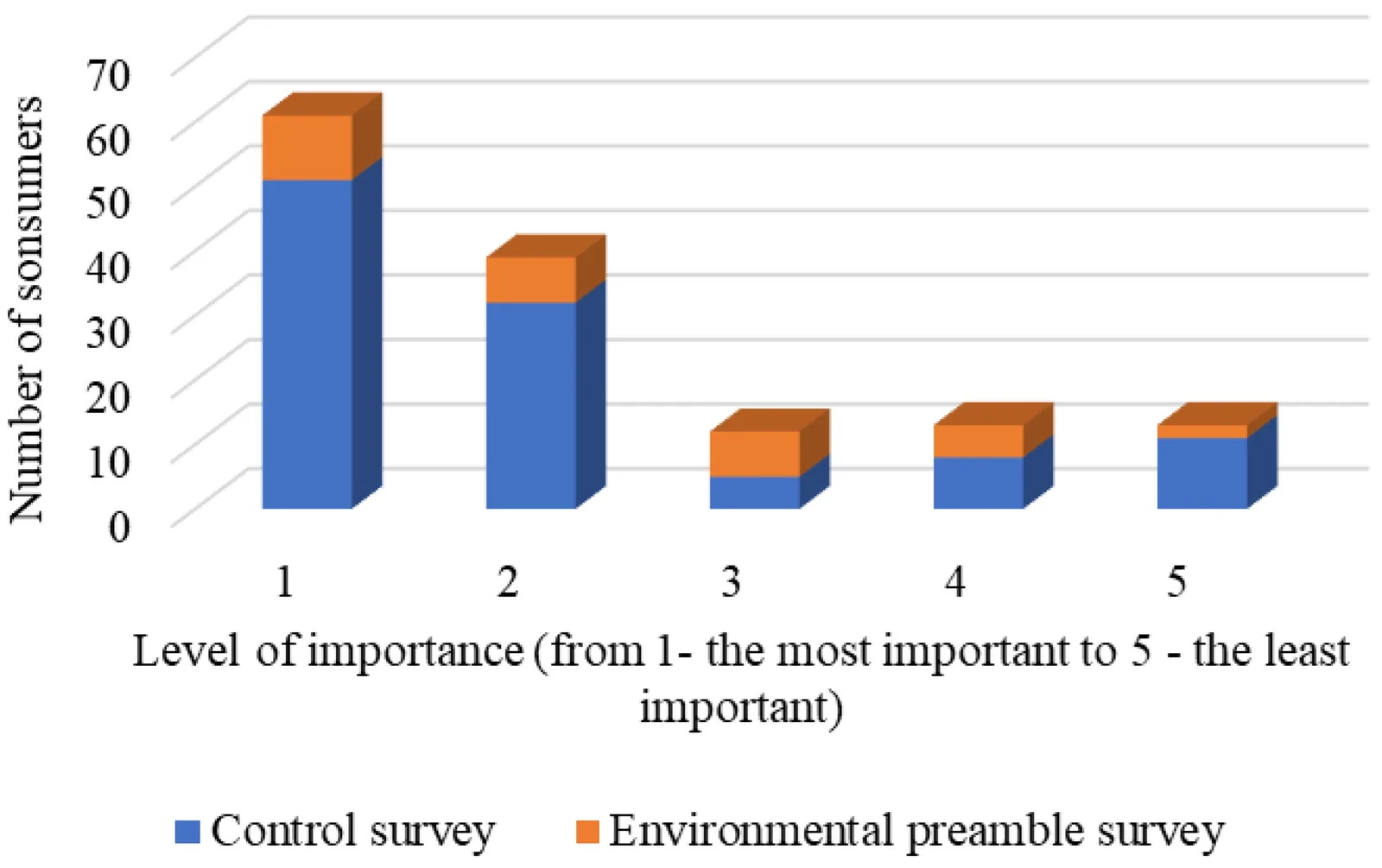
Consumers’ answers regarding the level of importance when comparing control and environment surveys.

Across the chi-square analysis, the control sample and informed groups of consumers showed that informed individuals assigned significantly different importance to the health and environmental aspects. These results are in accordance with the study of [59], where consumers emphasized the health and environmental aspects of beef production. Furthermore, in the study of [60], the information multiverse surrounding meat has a significant impact on meat consumers. On the other hand, consumers’ responses about the importance of the nutritional aspect, when they were directly informed that consuming meat has nutritional benefits, were not significantly different (Table 5). These results are shown in Figure 2, Figure 3 and Figure 4 and might be caused by the lack of knowledge about the nutritional benefits of meat or uncertainty about its benefits due to previous negative attitudes. Furthermore, one explanation can be that consumers pay more attention to information cues such as the expiration date, species name, weight and price than nutritional information [61].

Providing information on the health and environmental benefits of beef consumption could be an advantage for meat producers, as these concerns are commonly pronounced among consumers [24,56]. However, this should be carefully promoted, as health benefits are hard to use legally as a promotional strategy [62].

### 3.3. Practical Implication of the Study

The health benefits of meat consumption should be carefully investigated and promoted by interested parties, since there is increasing research on reducing animal-product consumption and there is a growing focus on interventions—actions taken by individuals, businesses, or governments—which incorporate health and environmental factors [12,63,64]. Furthermore, education about the health, environmental, socio-political, or animal welfare consequences of eating meat can reduce intended meat consumption, but there is little evidence on whether this approach influences actual behavior [63].

Nowadays, there are a lot of strategies to reduce meat consumption [65]. For instance, it was explored that the average Danish adult diet can be improved by an increase in the content of fruit and vegetables, whole grain, fish and fats from vegetable sources instead of animal sources and a decrease in red meat (beef, pork and lamb) [20]. This implies a need for future studies and for all people and organizations in meat supply chains to focus on the importance of strategies of promoting meat consumption. Furthermore, all parties in meat supply chains, including meat producers, retailers, governmental agencies and social media workers (influencers), need to use adequate and correct sources of information about meat consumption and related aspects. Furthermore, the promotion of the scientifically confirmed health, environmental and nutritional aspects of beef can override the effects of negative environmental and health perceptions of beef [60].

## 4. Conclusions

The study indicates that consumers are influenced by health and environmental information, whereas this was not the case with nutritional information. Consumers believed that mitigation of the risk of colorectal cancer and antibiotic residues are the most important attitudes of human health regarding beef consumption. Although consumers have strong positive attitudes about meat as a great source of proteins, vitamins and minerals, they show insignificant awareness of the importance of the nutritional aspects of beef when they were directly informed. Respondents who are supportive of reducing the environmental impact of livestock production tend to give the highest scores of importance for GHG emissions. On the other hand, they were not significantly concerned about climate change and livestock production’s negative effect.

The first limitation of the current study is the choice of socio-demographic characteristics, which does not include household income. Secondly, the data were collected in one country (Serbia) and this type of study is commonly based on a multinational level, especially when an online surveying method is used.

Future research can focus on investigating the impact of different ways of informing consumers about improvements in beef safety, quality, and healthiness. Segmenting consumers according to their awareness and interests in different beef production and consumption aspects may be a good practice for all stakeholders in the beef supply chain to make a systematic strategy of promoting certain information.

## Figures and Tables

**Table 1:** Demographic characteristics of participants (N = 956).

Characteristic	Control Survey	Health Preamble	Nutritional Preamble	Environmental Preamble	Total
Gender					
Female	112 (46.09%)	114 (50.44%)	180 (72.00%)	135 (56.96%)	541 (56.59%)
Male	131 (53.91%)	112 (46.09%)	70 (28.81%)	102 (41.98%)	415 (43.41%)
Age					
18–29	38 (15.64%)	23 (10.18%)	72 (28.80%)	39 (16.46%)	172 (17.99%)
30–39	52 (21.40%)	56 (24.78%)	55 (22.00%)	48 (20.25%)	211 (22.07%)
40–49	63 (25.93%)	80 (35.40%)	49 (19.60%)	92 (38.82%)	284 (29.71%)
50–59	78 (32.10%)	67 (29.65%)	59 (23.60%)	32 (13.50%)	236 (24.69%)
60 and more	12 (4.94%)	0 (-)	15 (6.00%)	26 (10.97%)	53 (5.54%)
Education					
Primary school	28 (11.52%)	25 (11.06%)	5 (2.00%)	9 (3.80%)	67 (7.01%)
High school	41 (16.87%)	58 (25.66%)	53 (21.20%)	32 (13.50%)	184 (19.25%)
Career school	24 (9.88%)	52 (23.01%)	57 (22.80%)	60 (25.32%)	193 (20.19%)
Bachelor’s degree	61 (25.10%)	53 (23.45%)	61 (24.40%)	65 (27.43%)	240 (25.10%)
Master’s	42 (17.28%)	24 (10.62%)	36 (14.40%)	28 (11.81%)	130 (13.60%)
PhD degree	47 (19.34%)	14 (6.19%)	38 (15.20%)	43 (18.14%)	142 (14.85%)
Household					
(members)					
1	35 (14.40%)	28 (12.39%)	37 (14.80%)	32 (13.50%)	134 (14.02%)
2	92 (37.86%)	62 (27.43%)	75 (30.00%)	55 (23.21%)	287 (30.02%)
3	86 (35.39%)	32 (14.16%)	80 (32.00%)	76 (32.07%)	278 (29.08%)
4	25 (10.29%)	70 (30.97%)	44 (17.60%)	65 (27.43%)	204 (21.34%)
5 and more	5 (2.06%)	34 (15.04%)	14 (5.60%)	9 (3.80%)	62 (6.49%)
Habits of beef consumption					
Frequency					
Every day	64 (26.34%)	43 (19.03%)	63 (25.20%)	29 (12.24%)	199 (20.82%)
Once or twice a week	89 (36.63%)	71 (31.42%)	114 (45.60%)	109 (45.99%)	383 (40.06%)
Once or twice a month	83 (34.16%)	98 (43.36%)	73 (29.20%)	86 (36.29%)	340 (35.56%)
Never	7 (2.88%)	14 (6.19%)	0 (-)	13 (5.49%)	34 (3.56%)

**Table 2:** The Kaiser–Meyer–Olkin (KMO) and Cronbach’s Alpha results obtained from the exploratory factor analysis (EFA) for validity and reliability assessments of consumers’ attitudes and extracted factors (F1, F2, F3).

Attitude Label	Attitude Description	F1	F2	F3	F1	F2	F3	Mean ± SD
		Validity (KMO)			Reliability (Cronbach’s Alpha)			
A1	Moderate consumption of meat mitigates the risk of colorectal cancer	**0.86**			**0.95**			4.82 ± 0.80
A2	It is important that meat does not contains antibiotics’ residues	**0.81**			**0.91**			4.58 ± 0.72
A3	It is important that meat does not contains pathogenic microorganisms	**0.67**			**0.85**			4.74 ± 0.98
A4	It is important to eat healthy	0.35			0.21			3.21 ± 0.94
A5	Meat proteins have great positive effect on human health		**0.68**			**0.89**		4.52 ± 1.13
A6	Meat is a great source of vitamins and minerals		**0.66**			**0.96**		3.81 ± 0.84
A7	There are more positive sides of eating meat than negative ones	0.38			0.35			3.07 ± 1.12
A8	Livestock is a great consumer of water							2.81 ± 0.93
A9	Livestock is a great consumer of energy							4.72 ± 0.87
A10	Livestock is a great consumer of fossil fuels							2.89 ± 0.78
A11	Livestock generate great amounts of organic waste							3.57 ± 0.73
A12	Livestock generate great amounts of inert waste		0.33			0.21		2.05 ± 1.12
A13	Livestock generate great amounts of hazardous waste		0.30			0.38		1.89 ± 1.04
A14	Livestock generate great amounts of waste waters		0.26			0.36		2.08 ± 0.83
A15	Livestock cause soil pollution							4.24 ± 0.79
A16	Livestock cause water pollution	0.21			0.11			3.87 ± 0.68
A17	Livestock cause air pollution		0.12			0.15		4.23 ± 1.14
A18	Livestock cause degradation of habitats		0.33			0.28		3.21 ± 1.22
A19	Livestock are a great source of greenhouse gas emissions			**0.63**			**0.85**	4.87 ± 0.98
A20	Livestock negatively affect climate change			**0.70**			**0.91**	3.94 ± 0.93

Note: Bolded values are significant for the appropriate factor (*p* < 0.05).

**Table 3:** Clusters based on the total number of consumers.

Clusters	Conscious About Health (Cluster 1)	Conscious About Nutrition (Cluster 2)	Concerned About Environment (Cluster 3)
Respondents	379 (39.6%)	340 (35.6%)	237 (24.8%)
Gender (%)			
Female	221 (58.3%)	146 (43%)	76 (32%)
Male	158 (41.7%)	194 (57%)	161 (68%)
Age (%)			
18–29	24 (6.4%)	154 (45.2%)	40 (17%)
30–39	52 (13.8%)	31 (9.2%)	23 (9.8%)
40–49	39 (10.3%)	52 (15.2%)	129 (54.5%)
50–59	192 (50.8%)	60 (17.6%)	22(9.2%)
60 and more	71 (18.7%)	44 (12.8%)	23 (9.5%)
Education (%)			
Primary school	46 (12.1%)	29 (8.5%)	46 (19.2%)
High school	18 (4.8%)	63 (18.4%)	8 (3.5%)
Career school	32 (8.4%)	39 (11.6%)	96 (40.5%)
Bachelor’s degree	62 (16.3%)	164 (48.2%)	31(13.2%)
Master’s	47 (12.3%)	18 (5.2%)	26 (11%)
PhD degree	175 (46.1%)	28 (8.1%)	30 (12.6%)
Mean ± SD			
A1 Moderate consumption of meat mitigates the risk of colorectal cancer	3.42 ± 0.79 ^a^	3.04 ± 1.18 ^b^	2.58 ± 0.91 ^c^
A2 It is important that meat does not contain antibiotic residues	4.02 ± 1.12 ^a^	3.18 ± 0.54 ^b^	2.63 ± 0.92 ^c^
A3 It is important that meat does not contain pathogenic microorganisms	3.42 ± 1.35 ^a^	3.64 ± 0.79 ^a^	3.87 ± 1.15 ^a^
A5 Meat proteins have a great positive effect on human health	3.76 ± 0.83 ^a^	4.24 ± 0.92 ^b^	3.14 ± 1.26 ^c^
A6 Meat is a great source of vitamins and minerals	3.25 ± 0.26 ^a^	3.91 ± 0.31 ^b^	2.77 ± 1.12 ^c^
A19 Livestock are a great source of greenhouse gas emissions	2.87 ± 1.02 ^a^	3.12 ± 0.54 ^b^	4.54 ± 1.35 ^c^
A20 Livestock negatively affect climate change	3.16 ± 1.21 ^a^	3.22 ± 1.04 ^a^	3.62 ± 0.78 ^a^

Note: Mean values ± standard deviations (SDs) were calculated using the raw data where a 5-point Likert scale was used as follows: Marks in the form of different superscript letters are based on the implemented Kruskal–Wallis H test and chi-square values. When different superscript letters are present, there is significant difference at the level 0.05 among clusters (*p* < 0.05).

**Table 4:** Chi-square values from the Kruskal–Wallis H test associated with the three clusters.

Test Variables ^b^							
	A1	A2	A3	A5	A6	A19	A20
Chi-Square ^a^	396.445	25.403	3.703	760.303	104.935	423.539	3.510
df	2	2	2	2	2	2	2
Asymptotic Sig.	0.000	0.000	0.157	0.000	0.000	0.000	0.173

Note: ^a^ *p* < 0.05; ^b^ Test variables for the Kruskal–Wallis H test are consumers’ attitudes extracted by EFA, while the grouping variable is the variable extracted by two-step cluster analysis.

**Table 5:** The mean ranks of the Friedman test for the indication of the relations among aspects.

Aspects	Mean Rank	C-H	C-N	C-E
Health	1.48 ^a^	0.111	0.098	0.085
Nutrition	2.04 ^a^	0.758	0.967	0.101
Environment	2.83 ^a^	0.324	0.111	0.007 *

Note: ^a^ *p* < 0.05; the Friedman test: chi-square value 41.759, df = 4. * *p* < 0.05 for the Wilcoxon signed-rank test. Abbreviations: C-H—“control survey vs. health preamble survey”; C-N—“control survey vs. nutritional preamble survey”; C-E—“control survey vs. environmental preamble survey.

## Data Availability

The original contributions presented in this study are included in the article/Supplementary Materials. Further inquiries can be directed to the corresponding author.

## Supplementary Materials

The following supporting information can be downloaded at: https://www.mdpi.com/article/10.3390/foods15172984/s1. Supplementary Material S1: Preambles of the Questionnaire.

## References

[1] FAO (2022). Meat Market Review.

[2] OECD, FAO (2021). OECD-FAO Agricultural Outlook 2021–2030.

[3] Stanisic S., Markovic V., Sarcevic D., Baltic M., Boskovic M., Popovic M., Kilibarda N. (2018). Being a vegetarian: Health benefits and hazards. Meat Technol..

[4] Gleason C.B., White R.R. (2019). Beef species-ruminant nutrition cactus beef symposium: A role for beef cattle in sustainable U.S. food production1. J. Anim. Sci..

[5] Park M.K., Kim B.-G., Kang M.-C., Kim T.-K., Choi Y.-S. (2025). Distinctive volatile compound profile of different raw meats, including beef, pork, chicken, and duck, based on flavor map. Appl. Food Res..

[6] Grosso G., La Vignera S., Condorelli R.A., Godos J., Marventano S., Tieri M., Ghelfi F., Titta L., Lafranconi A., Gambera A. (2022). Total, red and processed meat consumption and human health: An umbrella review of observational studies. Int. J. Food Sci. Nutr..

[7] O’Keefe J.H., Cordain L. (2004). Cardiovascular disease resulting from a diet and lifestyle at odds with our Paleolithic genome: How to become a 21st-century hunter-gatherer. Mayo Clin. Proc..

[8] Gnagnarella P., Caini S., Maisonneuve P., Gandini S. (2018). Carcinogenicity of High Consumption of Meat and Lung Cancer Risk Among Non-Smokers: A Comprehensive Meta-Analysis. Nutr. Cancer.

[9] de Medeiros G.C.B.S., Mesquita G.X.B., Lima S.C.V.C., Silva D.F.d.O., de Azevedo K.P.M., Pimenta I.D.S.F., de Oliveira A.K.d.S.G., Lyra C.d.O., Martínez D.G., Piuvezam G. (2023). Associations of the consumption of unprocessed red meat and processed meat with the incidence of cardiovascular disease and mortality, and the dose-response relationship: A systematic review and meta-analysis of cohort studies. Crit. Rev. Food Sci. Nutr..

[10] Gross S., Waldrop M.E., Roosen J. (2021). How does animal welfare taste? Combining sensory and choice experiments to evaluate willingness to pay for animal welfare pork. Food Qual. Prefer..

[11] Chen C., Chaudhary A., Mathys A. (2019). Dietary Change Scenarios and Implications for Environmental, Nutrition, Human Health and Economic Dimensions of Food Sustainability. Nutrients.

[12] Graça J., Godinho C.A., Truninger M. (2019). Reducing meat consumption and following plant-based diets: Current evidence and future directions to inform integrated transitions. Trends Food Sci. Technol..

[13] Grundy E.A.C., Slattery P., Saeri A.K., Watkins K., Houlden T., Farr N., Askin H., Lee J., Mintoft-Jones A., Cyna S. (2022). Interventions that influence animal-product consumption: A meta-review. Future Foods.

[14] Djekic I., Tomasevic I. (2016). Environmental impacts of the meat chain—Current status and future perspectives. Trends Food Sci. Technol..

[15] FAO (2021). Indicator 10: Individual Average Daily Consumption of Meat.

[16] Caro D., Davis S., Bastianoni S., Caldeira K. (2014). Global and regional trends in greenhouse gas emissions from livestock. Clim. Change.

[17] IPCC (2014). Climate Change 2014: Mitigation of Climate Change.

[18] Lamri M., Djenane D., Gagaoua M. (2022). Goat meat consumption patterns and preferences in three provinces of Kabylia region in Algeria compared to other meat species: Results of an online survey. Meat Technol..

[19] Djekic I., Blagojevic B., Antic D., Cegar S., Tomasevic I., Smigic N. (2016). Assessment of environmental practices in Serbian meat companies. J. Clean. Prod..

[20] Mogensen L., Hermansen J.E., Trolle E. (2020). The Climate and Nutritional Impact of Beef in Different Dietary Patterns in Denmark. Foods.

[21] Blanc S., Massaglia S., Borra D., Mosso A., Merlino V.M. (2020). Animal welfare and gender: A nexus in awareness and preference when choosing fresh beef meat?. Ital. J. Anim. Sci..

[22] De Backer C.J.S., Hudders L. (2015). Meat morals: Relationship between meat consumption consumer attitudes towards human and animal welfare and moral behavior. Meat Sci..

[23] Kılıç İ., Bozkurt Z. (2013). The Relationship between Farmers’ Perceptions and Animal Welfare Standards in Sheep Farms. Asian-Australas. J. Anim. Sci..

[24] Grunert K.G., Sonntag W.I., Glanz-Chanos V., Forum S. (2018). Consumer interest in environmental impact, safety, health and animal welfare aspects of modern pig production: Results of a cross-national choice experiment. Meat Sci..

[25] Garzillo J.M.F., Poli V.F.S., Leite F.H.M., Steele E.M., Machado P.P., Louzada M.L.d.C., Levy R.B., Monteiro C.A. (2022). Food consumption in Brazil: Influence of beef on environmental impact and nutritional quality of the diet. Rev. De Saúde Pública.

[26] Coffey A.A., Lillywhite R., Oyebode O. (2023). Meat versus meat alternatives: Which is better for the environment and health? A nutritional and environmental analysis of animal-based products compared with their plant-based alternatives. J. Hum. Nutr. Diet..

[27] Wang B., Shen C., Cai Y., Liu D., Gai S. (2022). The purchase willingness of consumers for red meat in China. Meat Sci..

[28] Kantono K., Hamid N., Ma Q., Chadha D., Oey I. (2021). Consumers’ perception and purchase behaviour of meat in China. Meat Sci..

[29] IARC (2015). IARC Monographs Evaluate Consumption of Red Meat and Processed Meat.

[30] Grunert K.G., Verbeke W., Kügler J.O., Saeed F., Scholderer J. (2011). Use of consumer insight in the new product development process in the meat sector. Meat Sci..

[31] Troy D.J., Kerry J.P. (2010). Consumer perception and the role of science in the meat industry. Meat Sci..

[32] Magalhaes D.R., Campo M.d.M., Maza M.T. (2021). Knowledge, Utility, and Preferences for Beef Label Traceability Information: A Cross-Cultural Market Analysis Comparing Spain and Brazil. Foods.

[33] Kim S.-Y., Maeng M.H. (2025). Association of Meat Attachment with Intention to Reduce Meat Consumption Among Young Adults: Moderating Role of Environmental Attitude. Nutrients.

[34] Cobern W., Adams B.A. (2020). Establishing survey validity: A practical guide. Int. J. Assess. Tools Educ..

[35] Yamane T. (1973). Statistics: An Introductory Analysis.

[36] Israel G.D. (2003). Determining Sample Size.

[37] Djekic I., Bozickovic I., Djordjevic V., Smetana S., Terjung N., Ilic J., Doroski A., Tomasevic I. (2021). Can we associate environmental footprints with production and consumption using Monte Carlo simulation? Case study with pork meat. J. Sci. Food Agric..

[38] Djekic I., Tomasevic I., Luetz J.M., Ayal D. (2021). Impact of Animal Origin Food Production on Climate Change and Vice Versa: Analysis from a Meat and Dairy Products Perspective. Handbook of Climate Change Management: Research, Leadership, Transformation.

[39] Tomasevic I., Solowiej B.G., Djordjevic V., Vujadinovic D., Djekic I. (2021). Attitudes and beliefs of Eastern European meat consumers–a review. IOP Conf. Ser. Earth Environ. Sci..

[40] Rajic S., Simunovic S., Djordjevic V., Raseta M., Tomasevic I., Djekic I. (2022). Quality Multiverse of Beef and Pork Meat in a Single Score. Foods.

[41] Kaiser H.F. (1974). An index of factorial simplicity. Psychometrika.

[42] Rajic S., Đorđević V., Tomasevic I., Djekic I. (2022). The role of food systems in achieving the sustainable development goals: Environmental perspective. Bus. Strategy Environ..

[43] Mašek J., Štefancová V., Mazanec J., Juránková P. (2023). The Classification of Application Users Supporting and Facilitating Travel Mobility Using Two-Step Cluster Analysis. Mathematics.

[44] Kallas Z., Realini C.E., Gil J.M. (2014). Health information impact on the relative importance of beef attributes including its enrichment with polyunsaturated fatty acids (omega-3 and conjugated linoleic acid). Meat Sci..

[45] Sakowski T., Grodkowski G., Gołebiewski M., Slósarz J., Kostusiak P., Solarczyk P., Puppel K. (2022). Genetic and Environmental Determinants of Beef Quality—A Review. Front. Vet. Sci..

[46] Verain M.C.D., Sijtsema S.J., Dagevos H., Antonides G. (2017). Attribute Segmentation and Communication Effects on Healthy and Sustainable Consumer Diet Intentions. Sustainability.

[47] Vainio A., Irz X., Hartikainen H. (2018). How effective are messages and their characteristics in changing behavioural intentions to substitute plant-based foods for red meat? The mediating role of prior beliefs. Appetite.

[48] Kwasny T., Dobernig K., Riefler P. (2022). Towards reduced meat consumption: A systematic literature review of intervention effectiveness, 2001–2019. Appetite.

[49] Jayalakshmi K., Paramasivam M., Sasikala M., Tamilam T., Sumithra A. (2017). Review on antibiotic residues in animal products and its impact on environments and human health. J. Entomol. Zool. Stud..

[50] Baltic M., Janjic J., Popovic M., Baltic T., Jankovic V., Starcevic M., Sarcevic D. (2018). Meat in traditional Serbian cuisine. Sci. J. Meat Technol..

[51] Chaudhary A., Tremorin D. (2020). Nutritional and Environmental Sustainability of Lentil Reformulated Beef Burger. Sustainability.

[52] Miller L.M.S., Cassady D.L. (2015). The effects of nutrition knowledge on food label use. A review of the literature. Appetite.

[53] Hong X., Li C., Bai J., Gao Z., Wang L. (2021). Chinese consumers’ willingness-to-pay for nutrition claims on processed meat products, using functional sausages as a food medium. China Agric. Econ. Rev..

[54] Hendrie G.A., Brindal E., Baird D., Gardner C. (2013). Improving children’s dairy food and calcium intake: Can intervention work? A systematic review of the literature. Public Health Nutr..

[55] Djekic I., Sanjuán N., Clemente G., Jambrak A.R., Djukić-Vuković A., Brodnjak U.V., Pop E., Thomopoulos R., Tonda A. (2018). Review on environmental models in the food chain—Current status and future perspectives. J. Clean. Prod..

[56] Verbeke W., Pérez-Cueto F.J., Barcellos M.D., Krystallis A., Grunert K.G. (2010). European citizen and consumer attitudes and preferences regarding beef and pork. Meat Sci..

[57] Navarrete-Molina C., Meza-Herrera C.A., Herrera-Machuca M.A., Lopez-Villalobos N., Lopez-Santos A., Veliz-Deras F.G. (2019). To beef or not to beef: Unveiling the economic environmental impact generated by the intensive beef cattle industry in an arid region. J. Clean. Prod..

[58] Li X., Jensen K.L., Clark C.D., Lambert D.M. (2016). Consumer willingness to pay for beef grown using climate friendly production practices. Food Policy.

[59] Żakowska-Biemans S., Pieniak Z., Gutkowska K., Wierzbicki J., Cieszyńska K., Sajdakowska M., Kosicka-Gębska M. (2017). Beef consumer segment profiles based on information source usage in Poland. Meat Sci..

[60] Djekic I., Rajic S., Dordevic V., Lazic I.B., Jovanovic J., Simunovic S., Terjung N., Volker H., Tomasevic I. (2024). Halo effect of persuasive messages towards pork. Fleischwirtschaft.

[61] Grunert K.G., Wills J.M. (2007). A review of European research on consumer response to nutrition information on food labels. J. Public Health.

[62] Stampa E., Schipmann-Schwarze C., Hamm U. (2020). Consumer perceptions, preferences, and behavior regarding pasture-raised livestock products: A review. Food Qual. Prefer..

[63] Bianchi F., Dorsel C., Garnett E., Aveyard P., Jebb S.A. (2018). Interventions targeting conscious determinants of human behaviour to reduce the demand for meat: A systematic review with qualitative comparative analysis. Int. J. Behav. Nutr. Phys. Act..

[64] Taufik D., Verain M.C.D., Bouwman E.P., Reinders M.J. (2019). Determinants of real-life behavioural interventions to stimulate more plant-based and less animal-based diets: A systematic review. Trends Food Sci. Technol..

[65] Harguess J.M., Crespo N.C., Hong M.Y. (2020). Strategies to reduce meat consumption: A systematic literature review of experimental studies. Appetite.

