# Supplementary material for: Consumer Attitudes Towards Health, Nutritional and Environmental Concerns of Beef Consumption by Confronting Different Information
Source: Foods. 2026 Aug 25;15(17):2984. doi: 10.3390/foods15172984 (PMC13565603; doi:10.3390/foods15172984)
Supplement: Supplementary file 1 [file foods-15-02984-s001.zip › foods-4499984-supplementary.pdf]

Supplementary Material S1: Preambles of the Questionnaire

Health preamble

In 2015, the International Agency for Research on Cancer (IARC) of the World Health Organization (WHO) labeled red meat as a Group 2A carcinogen, meaning an agent that probably causes cancer in humans. An IARC Monographs Working Group used epidemiological evidences and other relevant data to reach conclusions of carcinogenic effect of the red meat and meat products consumption. In 2019, based on the Global Burden Disease (GBD) data, a diet high in red meat was responsible for 0.9 million deaths and 23.9 million disability-adjusted life years (DALYs) worldwide. From 1990 to 2019, the total deaths and DALYs attributable to a diet high in red meat increased by over 50%. Beside cancerogenic effects, the overuse and misuse of antibiotics in the meat industry is known. The Food and Drug Administration (FDA) estimates that animal agriculture accounts for 70 % of all U.S. antibiotic use. However, this is not the end of potential hazards of meat consumption. The importance of meat-borne pathogens to global disease transmission and food safety is significant for public health. Consuming contaminated meat or meat products with drug-resistant bacteria is a severe public health hazard.

Nutritional preamble

It was reported by the Food and Agriculture Organization of the United Nations (FAO) that meat, eggs and milk offer crucial sources of needed nutrients, such as protein, fats and carbohydrates and micro-nutrients, which cannot easily be obtained from plant-based foods. High quality protein, a number of essential fatty-acids, iron, calcium, zinc, selenium, Vitamin B12, choline and bioactive compounds like carnitine, creatine, taurine are provided by foods from terrestrial animals and have important health and developmental functions. Protein availability from beef, pork, poultry, and sheep meat is projected to grow 5.9%, 13.1%, 17.8% and 15.7% respectively by 2030.

Environmental preamble

According to FAO data, 14.5% of all human-caused greenhouse gas emissions are attributable to livestock farming, an industry that emits not only carbon dioxide (CO_2), but also methane (CH_4) and nitrous oxide (N_2O) — two gases considered to play a similar role to CO_2 in driving global warming. Most emissions in livestock farming result from feed production (58%) and are released during animals' digestive processes (31%); ruminants such as cattle, sheep and goats produce large quantities of methane. Processing and transport account for sizable share of greenhouse gas emissions (7%), as well, as does the storage of manure (4%). Furthermore, slaughterhouses are the top emitting industry of phosphorus, a mineral that can asphyxiate fish by triggering explosive growth of algae and aquatic plants in water. They are also huge emitters of nitrogen, which can have a similar impact. This can be caused by releasing of waste waters, especially when it is known that meat industry waste waters are 5 to 10 times as strong as domestic sewage and contain much higher nitrogen, phosphorus and grease concentrations. Moreover, the application of pig manure has led to a certainty extend pollution of copper and zinc in soil, which becomes severity in the release of heavy metals in plantation.

SuppInfo S2: The preambles that were used for health preamble survey, nutritional preamble survey and environmental preamble survey, respectively. The control survey was given to consumers without preamble.
